# Investigations of the voltage-gated Ca^++^ channels and phosphodiesterase enzyme inhibitory-like potential explain the medicinal use of *Otostegia fruticosa* in diarrhea and hypermotile gut

**DOI:** 10.3389/fphar.2026.1790239

**Published:** 2026-03-04

**Authors:** Najeeb Ur Rehman, Mohd Nazam Ansari, Nasser Abdulaziz Alalaiwah, Eyad Omar Alotibi, Aman Karim, Tiegist Bahta, Khalil Y. Abujheisha, Muhammad Noman

**Affiliations:** 1 Department of Pharmacology and Toxicology, College of Pharmacy, Prince Sattam Bin Abdulaziz University, Al-Kharj, Saudi Arabia; 2 Department of Biological Sciences, National University of Medical Sciences Rawalpindi, Rawalpindi, Pakistan; 3 Department of Pharmacy, College of Health Sciences, Aksum University, Aksum, Ethiopia; 4 Department of Basic Medical Science, Faculty of Medicine and Health Science, Palestine Polytechnic University, Hebron, Palestine; 5 Department of Pharmacy Quaid-i-Azam University Islamabad, Islamabad, Pakistan

**Keywords:** antispasmodic, Ca^++^ channel blocker, *Otostegia fruticosa*, phosphodiesterase inhibitor, verapamil

## Abstract

**Background:**

The leaves of *Otostegia fruticosa* have been used in traditional medical systems to treat diarrhea and gut spasms. In this study, we evaluate the possible gut inhibitory roles of the crude extract of *O. fruticosa* using mice for *in vivo*, rabbits for *ex vivo*, and selected enteric pathogenic bacteria for *in vitro* assays.

**Methods:**

Castor-oil-induced diarrhea was used to assess the diarrheal protection of the extract in mice, while CaCl_2_-induced excitatory concentration–response curves (CRCs) and isoprenaline-induced inhibitory CRCs were constructed for isolated rabbit intestines to explore Ca^++^ channel blockade and phosphodiesterase (PDE) inhibitory-like pathways, respectively. Moreover, the antibacterial activity of the extract was tested against selected bacteria.

**Results:**

The extract protected mice from castor-oil-mediated diarrhea significantly compared to the saline control group at doses of 200 and 400 mg/kg. In the isolated jejunum, the extract inhibited both spontaneous and high K^+^-depolarized contractions at comparable concentrations in a dose-dependent manner (0.01–1 mg/mL) similar to papaverine, which is a dual inhibitor of the PDE enzymes and L-type Ca^++^ channels. The indirect functionality of the papaverine-like dual inhibitory actions of the extract was confirmed when pretreatment with the crude extract displaced the Ca^++^ excitatory CRCs to the right with suppression of the maximum response, similar to verapamil; moreover, the PDE inhibitory effect was authenticated by a leftward shift in the isoprenaline-induced inhibitory CRCs. The extract showed bactericidal activity with a resultant minimum inhibitory concentration (MIC) of 550 μg/mL against *Escherichia coli*, *Shigella sonnei*, and *Salmonella typhimurium,* whereas the extended-spectrum β-lactamase-producing *E. coli* strain was found to be sensitive to a higher concentration of the extract (MIC of 675 μg/mL).

**Conclusion:**

The present study is a pilot report on the detailed pharmacodynamics of the antispasmodic effects of the crude extract of *O*. *fruticosa,* with possible dual inhibition of the Ca^++^ channels and PDE-like effects, which provides a sound basis for its medicinal usage in hyperactive gut disorders. The *O. fruticosa* extract was further demonstrated to be effective against both enteric and non-enteric pathogens, which might support its use in the treatment of infectious diarrhea.

## 1 Introduction

Infectious and non-infectious diarrhea are considered to be some of the most prevalent causes of morbidity and mortality globally (GBD, 2016). In developed countries, the prevalence of chronic diarrhea ranges from 1% to 5% of the total adult population (Carrasco-Labra et al., 2019). Diarrhea is defined as the excessive loss of water and electrolytes by either an increase in the frequency of defecation or deformed stool episodes of three or more times a day (Tadesse et al., 2014; World Health Organization, 2017). The etiology of this disease is multifactorial, with possible contributions from varied factors like enteric infections (Tribble, 2017), food sensitivities, intestinal motility disorders (Anbazhagan et al., 2018), alcohol intake (Schiller, 2012), weak absorption of bile salts (Barkun et al., 2013), and/or the use of certain medications like β-blockers as well as antimicrobial, antineoplastic, antiretroviral, oral hypoglycemic, non-steroidal anti-inflammatory, and gastric acid lowering agents (Philpott et al., 2014). Appropriate assessment and therapeutic management of this condition is quite challenging as its diagnosis is not narrow, while differentiation between organic and functional causes of its etiology is another challenge (Burgers et al., 2020). The primary treatment of diarrhea is focused on patient rehydration, which is the most common form of non-pharmacological management for symptomatic relief of the discomforts associated with hyperkinetic bowel movements (Greenland et al., 2016; Schiller, 2017; Nalin and Cash, 2018). Contrarily, the pharmacological management of diarrhea is non-specific and mainly focused on reducing the clinical symptoms through the use of multiple classes of drugs, such as antisecretory and antimotility agents, probiotics, enkephalinase blockers, bismuth salts, and α2-adrenergic receptor agonists (Cheng, 2016; Szymaszkiewicz et al., 2019). However, the use of these pharmacological agents is limited by various side effects like gastric cramps, intestinal distension, oral dryness, constipation, nausea, and vomiting (Wu and Juurlink, 2017). Similarly, loperamide is a popular opioid agonist used to treat diarrhea, which causes respiratory depression and paralytic ileus in children that limit its usage in clinical practice (Zarghami and Rezapour, 2017). Despite the abundance of conventional medications, medicinal plants as alternative therapeutic agents have been shown to offer significant preventative and therapeutic effects. The crude extracts of these medicinal plants as well as their isolated pure compounds, semisynthetic derivatives, and/or synthetic compounds are the most readily available drugs in practice today (Wright, 2019). Given this evidence-based practice, plants with medicinal properties are assumed as ideal starting points for guiding the development of new drugs against gastrointestinal disorders like diarrhea (Shen, 2015; Ahn, 2017). Additionally, Costa et al. (2015) recommend the addition of medicinal plants to foodstuff owing to their antimicrobial benefits. Another study by Cheesman et al. (2017) showed that combinations of antibiotics and natural antimicrobial substances from plant sources could combat multidrug-resistant bacteria.

*Otostegia fruticosa* is a type of shrubby bush lily that is widely distributed in Saudi Arabia, Yemen, Ethiopia, and Palestine and is commonly known as “sharm” in the Arabian peninsula (Adgaba et al., 2017) and “sasa” in northern Ethiopia (Andemariam, 2010). The medicinal uses of this plant have long been recorded in various traditional healing systems. For instance, in Saudi Arabia, the infusion of *O. fruticosa* flowers has been used as a remedy against sunstroke (Rahman et al., 2004). In Ethiopia, Kidane et al. (2013) documented the use of its leaves by the Tigray people as a mosquito repellent. In Yemeni folkloric medicine, the plant is known for its medicinal effects on both humans and animals, i.e., as an antiparalytic to humans and an anti-inflammatory for application to animal eyes (Ali et al., 2017; Mothana et al., 2011). Al-Musayeib et al. (2000) isolated several compounds from the aerial parts of the plant, including labdane diterpenes, otostegin A, otostegin B, and 15-epi-otostegin B. Aside from the leaf extract of *O. fruticosa*, its essential oil has been tested for antibacterial (Aboutabl et al., 1995), antifungal (Ali et al., 2017), and antioxidant (Mothana et al., 2011) activities. In a study on animals, Bahta et al. (2020) explored the analgesic and anti-inflammatory properties of its chloroform fraction. The juice of *O. fruticosa* leaves is commonly used to treat diarrhea in children in the Ethiopian traditional healing system (Meragiaw et al., 2016). Despite multiple traditional reports of its effectiveness, there are no detailed scientific studies to date on the potential of the leaf extract as an antidiarrheal and antispasmodic agent in animal studies. Therefore, the present work may be considered a detailed pilot study on exploring the potential of *O*. *fruticosa* extracts in the treatment of diarrhea and hyperactive gut disorders.

## 2 Materials and methods

### 2.1 Chemicals

The following analytical grade chemicals were obtained from Sigma (St. Louis, MO, United States) and used as received: carbamylcholine (CCh), loperamide, acetylcholine (ACh) perchlorate, isoprenaline, verapamil, and papaverine. The reagents (salts) used to prepare the physiological buffer solution (Tyrode’s buffer) are as follows: potassium chloride (Sigma), calcium chloride, glucose, magnesium sulfate, potassium dihydrogen phosphate, sodium bicarbonate, and sodium chloride (Merck, Germany). Castor oil was obtained from a local pharmacy in Al-Kharj. The Mueller–Hinton broth (MHB, Scharlab) and Muller–Hinton agar (MHA, Scharlab) were used for the antibacterial assays, and MacConkey agar (Oxoid) was used to cultivate the tested bacteria. All chemicals used in these experiments were of analytical grade except the diarrhea-inducing agent (castor oil) procured from a local pharmacy in Al-Kharj.

### 2.2 Plant material collection and extraction

The leaves of *O. fruticosa* were collected from Wukro Kilteawaelo town in the Tigray region of Ethiopia. The samples were authenticated by Shamle Amelu, and the voucher specimen (no. 001) was deposited in the National Herbarium of the College of Natural and Computational Sciences, Addis Ababa University, Ethiopia.

The shade-dried plant material was powdered and macerated for 72 h in 70% ethanol and filtered, and this process was repeated three times. The combined filtrate was dried in an oven at 40 °C, and the aqueous ethanolic crude extract obtained was stored at −4 °C for further use.

### 2.3 Animals

Healthy male rabbits (2.5–3 kg) were procured from a nearby farm in Al-Kharj one day before the experiments, and Swiss albino mice (20–25 g) were obtained from the Animal Care Unit of the College of Pharmacy, Prince Sattam bin Abdulaziz University (PSAU). The mice were housed at an optimum temperature of 22 °C ± 1 °C and relative humidity of 55% ± 5% with 12-h equal light/dark cycles. The rabbits were fasted for 24 h by food restriction, followed by displacement euthanasia via a forceful blow to the back of the neck (Rehman et al., 2015). The ethical guidelines and instructions detailed in National Research Council (1996) were followed strictly while performing both the *in vivo* and *ex vivo* experiments. The assay protocols were approved by the Standing Committee of the Bioethics Research (SCBR) at PSAU (reference number SCBR-394/2024).

### 2.4 *In vivo* experiments

In this preventive screening study, 20 mice of both genders were randomly and equally allocated into four groups. Following 24 h of fasting, the mice in the first group were administered normal saline at 10 mL/kg once daily and were labeled as disease or negative control. After a pilot screening phase to identify the effective antidiarrheal dose, the second and third groups (test groups) were administered *O. fruticosa* crude extract at doses of 200 and 400 mg/kg (once daily), respectively. Loperamide (10 mg/kg once daily) was administered to the fourth group as a reference control (Ansari, 2020). One hour following the abovementioned treatments, the mice in all groups were challenged with castor oil (10 mL/kg, once daily), and each mouse was placed in a separate cage with a blotting sheet at the base. Six hours after castor oil administration, all the blotting sheets were checked individually by a blinded observer for the presence or absence of diarrheal spots (Jebunnessa et al., 2009).

All interventions were administered by oral gavage at an approximate volume of 0.2–0.3 mL to each mouse.

### 2.5 *Ex vivo* protocol

#### 2.5.1 Spasmolytic assay

The functional studies were based on slight modification of an earlier method reported by Shah et al. (2011). Following dissection of each rabbit, the middle part of the intestine (jejunum) was isolated carefully using a sharp scissor and immediately placed in the physiological buffer (Tyrode’s solution). Multiple tissue samples were obtained from each animal by cutting approximately 2–3 cm segments of the jejunum, and each tissue section was cleaned off gently from the adjacent mesenteries. The jejunal tissues were also cleaned of any internal fecal material by gently squeezing the tissue from the middle toward the ends using the blunt side of a forceps to avoid injuries. One end of each tissue section was attached via thread to a metal hook, while the other end was attached to an isotonic transducer integrated amplifier connected to the IOX software, emkaBath (France) (Ansari, 2020). Fresh Tyrode’s buffer was filled in 20-mL tissue baths bubbled with carbogen, and the temperature was maintained at 37 °C throughout the experiment. The millimolar composition of Tyrode’s solution was as follows: KCl, 2.68; NaCl, 136.9; MgCl_2_, 1.05; NaHCO_3_, 11.90; NaH_2_PO_4_, 0.42; CaCl_2_, 1.8; glucose, 5.55 (pH 7.4). A tension of 1 g was applied by rotating the transducer knob clockwise, and the tissues were left to stabilize for 45 min without the addition of any drug. The bath buffer was freshly replaced in 10-min intervals. ACh (0.3 µM) was added to the bath to obtain a stable band of the spontaneous contractions of the jejunum. The plant extract was added cumulatively to ensure a final bath concentration of 0.01–10 mg/mL to record the inhibitory effect on the spontaneous contractions. The responses observed upon addition of the crude extract or standard drug to the isolated tissue bath were quantified by dose in a cumulative manner. The observed relaxant effects of the extract/standard drug were quantified as percent decreases in the spontaneous contractions of the tissue preparation recorded immediately prior to addition of the test sample. Once the relaxation effect of the crude extract on spontaneous contraction was observed, additional experiments were conducted to explore the possible pharmacodynamics. Following the procedures in a previously reported study by Godfraind et al. (1986), the crude extract was tested against contractions elicited by high K^+^ (80 mM) to determine the possibility of Ca^++^ channel blockade (CCB). The final bath concentration of KCl (K^+^ >30 mM) was used to produce strong and sustained contractions in the isolated tissues via depolarization of multiple smooth muscles through opening of the voltage-gated Ca^++^ channels (L-type). The observed relaxant effects from cumulative addition of the plant extract on high-K^+^-induced contractions were expressed as percentages of the control contractile responses. Any substance that inhibits this sustained contraction is considered as a Ca^++^ channel blocker (Gilani et al., 1997). Another critical observation reported by Gilani et al. (2005) is that if the relaxation of a test material against spontaneous and high-K^+^-induced contractions is observed at comparable concentrations, then such materials may be considered as phosphodiesterase (PDE) inhibitors.

#### 2.5.2 Exploration of Ca^*++*^ channel antagonism

After recording the relaxing effects of the plant extract against sustained contractions induced by high K^+^ (KCl) concentrations, further confirmation of the CCB was explored by preincubating the jejunal tissues with the extract in an organ bath for 30 min in modified Ca^++^-free Tyrode’s solution containing EDTA (0.1 mM) to chelate the Ca^++^ from the tissue. Moreover, to ensure that the tissues are completely free of Ca^++^, the intracellular stores of Ca^++^ were depleted using the method reported by Blattner et al. (1978). In brief, the earlier Ca^++^-free solution of the tissue bath was replaced with a K^+^-rich (high K^+^) and Ca^++^-free Tyrode’s solution, as detailed in Shah et al. (2011). After the jejunal tissue was allowed to incubate for 60 min in this buffer, exogenously prepared Ca^++^ using CaCl_2_ was added to the tissue bath cumulatively at increasing concentrations to construct the control concentration–response curves (CRCs) for Ca^++^; these procedures were then repeated in the tissues pretreated with increasing concentrations of the extract. Tissues preincubated with verapamil were observed in parallel for comparison of the CCB effect.

#### 2.5.3 PDE inhibitory activity

Based on the preliminary findings for the crude extract at similar potency against spontaneous and high-K^+^-induced contractions in isolated jejunal preparations, the PDE-inhibitory-like potential of the extract was authenticated indirectly by isoprenaline-induced inhibitory CRCs. These curves were constructed against CCh-mediated contractions in the control and preincubated jejunal tissues for increasing concentrations of the extract. Shifting of the isoprenaline inhibitory CRCs to the left (lower concentrations) indicates the PDE-inhibitory-like action of the test sample, which was compared with the curves of the reference drug papaverine as a standard PDE inhibitor (Rang et al., 1999).

### 2.6 *In vitro* experiments

#### 2.6.1 Bacterial strains

The antibacterial effects of the crude extract were tested against reference strains from the American Type Culture Collection (ATCC) available from the Microbiology Laboratory at the College of Pharmacy, PSAU. The standard bacterial strains used were as follows: *Escherichia coli* (ATCC 11209), *Salmonella typhimurium* (ATCC 14025), and *Shigella sonnei* (ATCC 11060). In addition, we tested the clinical pathogen extended-spectrum beta-lactamase (ESBL)-producing *E. coli* isolated and identified by Dr. Khalil at King Khalid Hospital (research study IRB-18-477E). The tested strains were cultivated routinely in MacConkey agar and grown aerobically at 37 °C.

#### 2.6.2 Determination of minimum inhibitory concentration (MIC)

The antibacterial assays for the MICs were accomplished by the broth dilution method according to the guidelines of the Clinical and Laboratory Standards Institute (CLSI, 2016), with some modifications to the protocol as described by Duarte et al. (2015). Different dilutions were prepared ranging from 400 to 750 μg/mL, and a volume of 10 µL of the test bacteria was inoculated into each concentration. The MHB alone was tested as a sterile control, and untreated bacteria inoculated on MHB with 1% dimethyl sulfoxide (DMSO) were tested as the growth control. The antimicrobial effects of the test samples were measured in terms of the MICs, which indicate the lowest concentrations of the antimicrobial samples that inhibit visible microbial growth. Next, the minimum bactericidal concentration (MBC) of the crude extract was recorded by subculturing 10 µL from the broth dilution in MHA, which resulted in 100% killing of the microbes. All assays were carried out in duplicate to confirm the results.

### 2.7 Statistics

The data were expressed as mean ± standard error of the mean (SEM) of the extract and reference drugs, where n = number of repetitions of an assay. EC_50_ depicts the median effective concentration with 95% confidence interval (CI). The protective effects against diarrhea by the extract and loperamide were evaluated statistically by comparing the first, second, and third groups with the saline control group through the Chi-squared (χ^2^) test. The CRCs were analyzed by non-linear regression, and two-way ANOVA followed by Bonferroni’s post-hoc test correction or an unpaired *t*-test was used for multiple comparisons of the CRCs with the respective controls. The Statistical significance was considered only when *p* < 0.05.

All graphing, calculations, and statistical analyses were performed using GraphPad Prism 4 for Windows (GraphPad Software, San Diego, CA, United States).

## 3 Results

### 3.1 Percentage protection offered by test samples in the castor oil model

Mice that were preadministered the crude extract showed significant protection (*p* < 0.05) against castor-oil-induced diarrhea in a dose-dependent manner compared to the saline-treated group. Two out of five mice that received the lower dose of extract (200 mg/kg) resisted castor-oil-induced diarrhea with 40% protection, while the mice receiving the higher dose of 400 mg/kg showed 80% protection. The reference drug loperamide enabled 100% protection, as detailed in Table 1.

**TABLE 1:** Antidiarrheal activity of the crude extract of *Otostegia fruticosa* on castor-oil-induced (10 mL/kg) diarrhea in mice.

Treatment (once daily), dose (mg/kg)	No. of mice with diarrhea	% protection
Saline (10 mL/kg) + castor oil	5/5	0
*O. fruticosa* crude extract + castor oil 200 (mg/kg) + 10 (mL/kg) 400 (mg/kg) + 10 (mL/kg)	3*/5 1*/5	40 80
Loperamide (10 mg/kg) + castor oil	0**/5	100

*p < 0.05 and **p < 0.01 vs. saline + castor-oil-treated group (χ^2^-test).

### 3.2 Relaxant effects on spontaneous jejunal contractions

**FIGURE 1:**
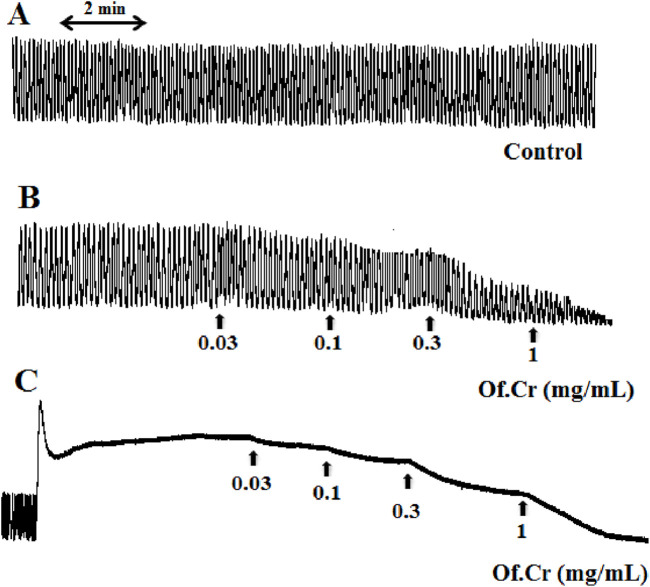
**(A)** Spontaneous contraction of the isolated rabbit jejunum and **(B)** relaxant effect of the crude extract of *Otostegia fruticosa* (Of.Cr) on spontaneous and **(C)** high-K^+^-induced (80 mM) contractions. The extract was added in increasing concentrations, and the values shown are the final concentrations in the tissue bath.

**FIGURE 2:**
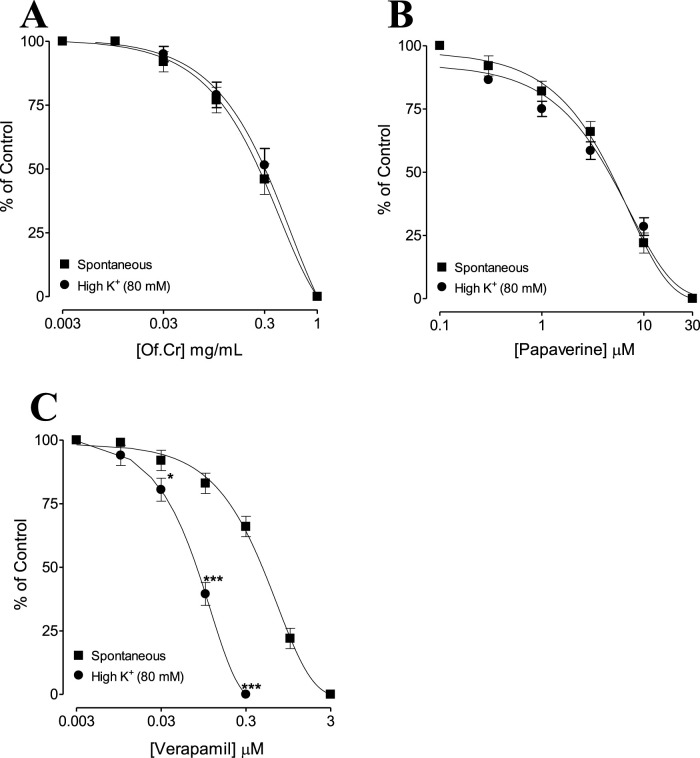
Concentration–response curves comparing the effects of **(A)** crude extract of *O. fruticosa* (Of.Cr) with **(B)** papaverine and **(C)** verapamil for inhibition of spontaneous and high-K^+^-induced contractions in isolated rabbit jejunum preparations. The values shown are mean ± standard error of the mean (SEM) for n = 4–5. *p < 0.05 and ***p < 0.001 (two-way ANOVA followed by Bonferroni’s post-hoc test correction or unpaired *t*-test).

Figure 1A shows the typical response of the spontaneously contracting rabbit jejunum; administration of the plant extract of in increasing concentrations relaxed the spontaneous contractile band of the jejunum (Figure 1B), with a recorded EC_50_ of 0.26 mg/mL (95% CI: 0.24–0.37, n = 6), as shown in Figure 2A. The reference drug papaverine showed an EC_50_ value of 6.64 µM (95% CI: 5.28–7.38, n = 6) (Figure 2B), and verapamil demonstrated an EC_50_ value of 1.52 µM (95% CI: 0.82–2.14, n = 6) with the same induced spontaneous relaxation (Figure 2C).

### 3.3 Effects of *O. fruticosa* extract and reference drugs on Ca^++^ channels

**FIGURE 3:**
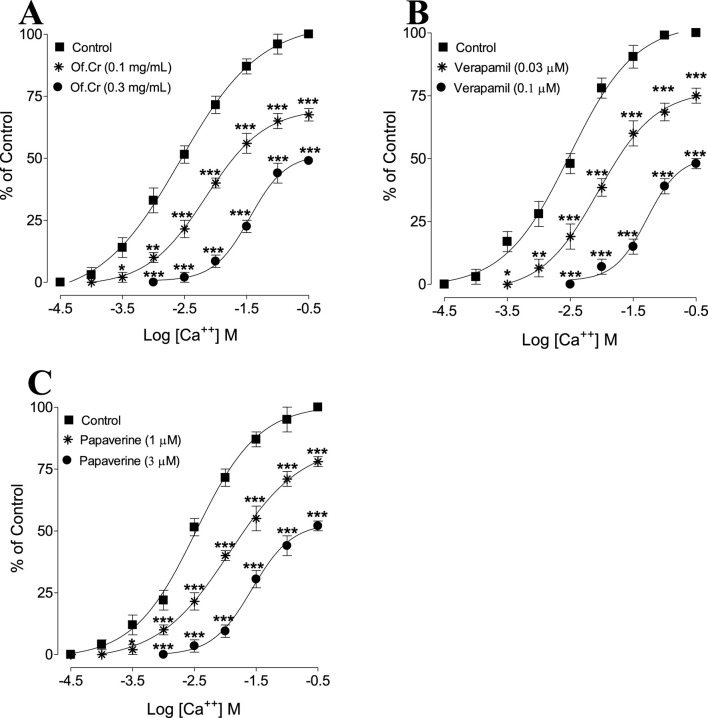
Concentration–response curves of CaCl_2_ (Ca^++^) in the absence and presence of increasing concentrations of the **(A)** crude extract of *O. fruticosa* (Of.Cr), **(B)** verapamil, and **(C)** papaverine in isolated rabbit jejunum preparations. The values shown are mean ± SEM for n = 4–5. *p < 0.05, **p < 0.01, and ***p < 0.001 show comparisons of the mean Ca^++^-mediated contractions in tissues pretreated with Of.Cr, verapamil, and papaverine with the respective means of Ca^++^-mediated contractions in the control (untreated) jejunal preparations (two-way ANOVA followed by Bonferroni’s post-hoc test correction or unpaired *t*-test).

Figure 1C shows a typical tracing of the effect of the crude extract against high-K^+^-induced contractions, where the extract reversed the sustained contraction; the results were similar for verapamil with a recorded EC_50_ of 0.29 mg/mL (95% CI: 0.26–0.39, n = 5) and the extract with an EC_50_ value of 0.16 µM (95% CI: 0.08–0.26, n = 5), as shown in Figures 2A,C. The verapamil-like (Figure 3B) CCB effects mediated by the extract at doses of 0.1 and 0.3 mg/mL were further authenticated by the rightward shift in the Ca^++^ curves along with E_max_ suppression (Figure 3A). Papaverine (1 and 3 µM) also exhibited suppression of E_max_ in the Ca^++^ CRC along with rightward deflection (Figure 3C).

### 3.4 PDE inhibitory effects of *O. fruticosa* extract and reference drugs

**FIGURE 4:**
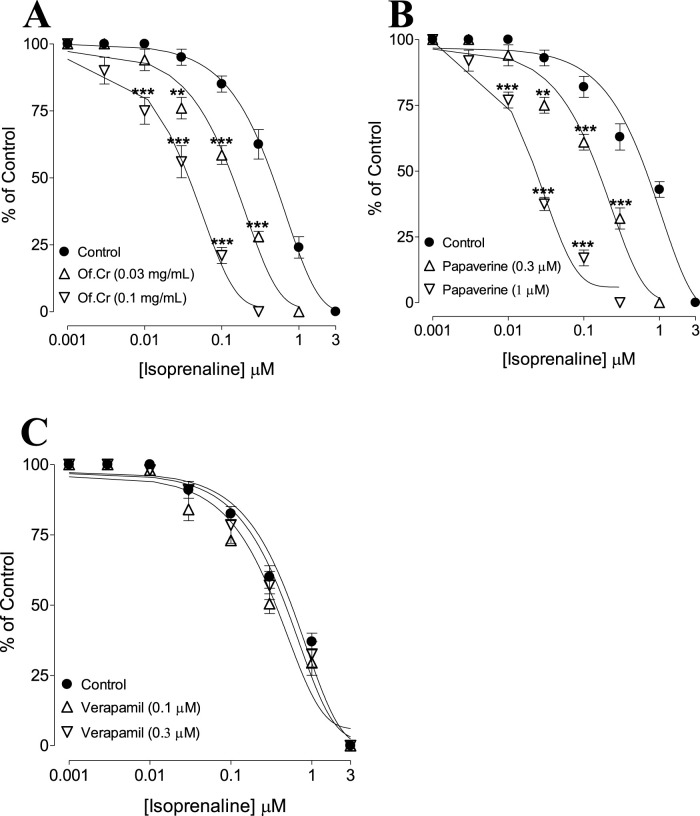
Inhibitory concentration–response curves of isoprenaline against carbamylcholine (CCh)-induced contractions in the absence and presence of different concentrations of the **(A)** crude extract of *O. fruticosa* (Of.Cr), **(B)** papaverine, and **(C)** verapamil in isolated rabbit jejunum preparations. The values shown are mean ± SEM for n = 4–5. **p < 0.01 and ***p < 0.001 show comparisons of the mean isoprenaline-mediated inhibitory effects in tissues pretreated with Of.Cr, papaverine, and verapamil with the respective means of isoprenaline-mediated inhibitory effects in the control (untreated) jejunal preparations (two-way ANOVA followed by Bonferroni’s post-hoc test correction or unpaired *t*-test).

Jejunal tissues preincubated with the extract (0.03 and 0.1 mg/mL) displaced the isoprenaline CRCs to the left (Figure 4A), indicating potentiating effects. The reference drug papaverine (0.3–1 μM) produced a similar leftward shift of the isoprenaline curves (Figure 4B), whereas verapamil did not affect the isoprenaline CRCs (Figure 4C).

### 3.5 Antibiotic susceptibility pattern of ESBL *E. coli*

In the present investigations, ESBL *E. coli* samples were used as a clinical pathogen. The antibiotic susceptibility pattern of ESBL *E. coli* was identified by Dr. Khalil at King Khalid Hospital (research study IRB-18-477E). The antibiotic susceptibility was tested using an automated microbiology testing system (Phoenix 100/BD), and the results were interpreted according to the Clinical and Laboratory Standard Institute (CLSI) guidelines. The ESBL *E. coli* sample was resistant to cefotaxime, ampicillin, amoxicillin/clavulanate, cefuroxime, ceftazidime, cefepime, and aztreonam.

#### 3.5.1 MIC and MBC determination

Table 2 illustrates the MIC and MBC values of the crude extract against the tested bacteria. There were no observable differences in sensitivity among the standard tested bacteria (MIC of 550 μg/mL); however, the ESBL *E. coli* showed more resistance owing to the higher recorded MIC of 675 μg/mL (Table 2).

**TABLE 2:** Minimum inhibitory and bactericidal concentrations (MIC and MBC) of the crude extract of *O. fruticosa* against the test bacteria.

Bacteria	MIC	MBC
*E. coli* (ATCC 11209)	550 μg/mL	575 μg/mL
*S. sonnei* (ATCC 11060)	550 μg/mL	575 μg/mL
*S*. *typhimurium (*ATCC 14025)	550 μg/mL	575 μg/mL
^a^ESBL *E. coli*	675 μg/mL	700 μg/mL

^a^ Clinical bacteria.

## 4 Discussion

Given the claims regarding the wide therapeutic potential of *O*. *fruticosa* in hyperactive gut disorders, such as stomach aches, diarrhea, and gut spasms (Bahta et al., 2020; Ansari et al., 2021), we tested the medicinal effects of the crude extract of this plant against diarrhea and gut spasms using the castor-oil-induced diarrhea model *in vivo* (Iwao and Terada, 1962) as well as the detailed mechanism(s) using isolated rabbit jejunum as the *ex vivo* model. Upon ingestion, castor oil is metabolized by the intestinal lipases in the body to release ricinoleic acid in the intestinal lumen. This not only causes strong contractions in the intestinal transverse and distal colon resulting in watery diarrhea (Croci et al., 1997) but also suppresses the Na^+^ and K^+^ ATPase activities to block sodium, chloride, and water absorption; elevate smooth muscles spasms; produce cytotoxic effects on the enterocytes; and result in intestinal fluid accumulation (Shahed-Al-Mahmud et al., 2020). Therefore, this model has been commonly used by our research group to screen unknown substances for possible antidiarrheal effects (Rehman et al., 2015). In the current study, mice that were preadministered 200 and 400 mg/kg of the plant extract experienced protective effects against diarrhea in a dose-mediated manner, similar to the positive reference drug loperamide that is used as a standard antidiarrheal agent. Upon observing the antidiarrheal responses of the extract *in vivo*, we planned further *ex vivo* experiments to explore the possible pharmacodynamics involved. The method reported by Bashir et al. (2011) was used to test and analyze the concentration-dependent antispasmodic potential of the extract in spontaneously contracting isolating rabbit jejunum in the *ex vivo* model. Interestingly, the extract enabled complete relaxation of the spontaneous contractions, thus exhibiting an antispasmodic effect supporting our *in vivo* study findings. Based on our previous observations, medicinal plants with antispasmodic properties commonly involve CCB (Rehman et al., 2015) and/or PDE enzyme inhibition (Khan et al., 2014). These reports encouraged us to test the extract on induced contractions using a high K^+^ concentration as a contractile agent (Gilani et al., 2013). The critical analysis of the patterns of the inhibitory CRCs of the extract against spontaneous and high-K^+^-induced contractions showed no significant difference (*p* > 0.05), similar to the effects of papaverine, which is a dual inhibitor of Ca^++^ channels and PDE (Rehman et al., 2022). However, the reference drug verapamil selectively exhibited higher potency against K^+^-depolarized tissues in comparison to its effects against the spontaneous jejunal contractions, and this pattern of relaxation has been reported earlier as CCBs (Fleckenstein, 1977). Relaxation of the K^+^ (>30 mM)-evoked spasms by any agent can be considered as CCB, as reported earlier by Hamilton et al. (1986); hence, to support and further confirm the CCB-like actions of the crude extract, the jejunal tissues were preincubated with the extract and stabilized in a Ca^++^-free buffer solution. When contractile CRCs of exogenously added Ca^++^ were achieved in the absence (control) and presence of the extract, deflection of the Ca^++^ CRCs to the right along with suppression of the maximum response (E_max_) were recorded similar to those of verapamil, thereby indirectly authenticating the CCB-like effects. When the extract was tested for possible inhibitory potential of PDE, we found a positive shift of the isoprenaline-induced inhibitory CRCs to the left, implying potentiating effects similar to those of papaverine, which is a referenced PDE inhibitor (Choo and Mitchelson, 1978; Sato et al., 2006). PDE present in the tissues converts active cAMP to its inactive form AMP to resist relaxation of smooth muscles (Smith et al., 2006). Moreover, Kaneda et al. (2005) have reported the relaxing effects of CCh-induced sustained spasms of smooth muscles, which we plan to explore in the future to further support and strengthen our present indirect findings of the PDE-inhibitory-like components in the crude extract of *O. fruticosa*. Apart from functional gut motility disorders, infectious agents can cause diarrhea and serve as an additional health burden (Palla et al., 2015). The antibiotic drugs available in the market have been found to be effective for managing infectious diarrhea (Farthing et al., 2013); however, there have been increasing reports on microbial resistance against these antibiotics (Kumarasamy et al., 2010), which have urged researchers to explore safer alternatives against these pathogens. Previously, our research group has reported the discovery of plant-derived antimicrobial agents (Khan et al., 2011) as well as animal-derived (Khan et al., 2008) and microbial (Iqbal et al., 2014) sources. Herein, we show that *O. fruticosa* extract has antimicrobial activities against selected pathogens like *E*. *coli*, *S. sonnei,* and *S. typhimurium* that cause enteric infections (Palla et al., 2015). An earlier study also reported the antibacterial activity and effectiveness of *O. fruticosa* essential oil against Gram-positive and -negative bacteria; this suggests that the constituents of the extract support its potential use against infectious diarrhea and may even serve as a source of new and better antimicrobial compounds for treating gastroenteritis (Aboutabl et al., 1995).

## 5 Conclusion

In this study, we show that the crude ethanolic extract of *O*. *fruticosa* possesses antidiarrheal and antispasmodic activities that are possibly mediated by voltage-gated CCB and PDE inhibition; however, additional mechanisms cannot be ignored. The findings of this study coupled with the antibacterial effects against select enteric and non-enteric pathogens may support the potential use of *O. fruticosa* extract in the treatment of infectious diarrhea. We also recommend further electrophysiological investigations and imaging experiments of the voltage-gated Ca^++^ channel subtypes, specific PDE inhibitory pathways, and molecular interactions of the metabolites with the binding sites as future directions of exploration.

## Data Availability

The raw data supporting the conclusions of this article will be made available by the authors without undue reservation.
